# Protocol for disseminating an evidence-based fall prevention program in community senior centers: evaluation of translatability and public health impact via a single group pre-post study

**DOI:** 10.1186/1748-5908-9-63

**Published:** 2014-05-26

**Authors:** Fuzhong Li, Peter Harmer

**Affiliations:** 1 Oregon Research Institute, 1776 Millrace Dr, Eugene, OR 97403, USA; 2 Department of Exercise Science, Willamette University, Salem, OR 97310, USA

**Keywords:** Falls, Community-dwelling older adults, Falls prevention, Tai Ji Quan

## Abstract

**Background:**

Falls are the leading cause of injury death in older adults and present a significant public health problem and a major burden to healthcare. Although there is sufficient evidence from randomized controlled trials to indicate that exercise can prevent falls in older people, few effective, evidence-based fall prevention programs exist in community practice. Thus, there is a pressing need to translate and disseminate evidence-based exercise programs to community providers that serve older adults at increased risk of falling. The current study addresses this public health need by disseminating the evidence-based Tai Ji Quan: Moving for Better Balance (TJQMBB) program through community senior centers.

**Methods/Design:**

The study uses a single-group design in which the TJQMBB program is being delivered to community-dwelling older adults through collaboration with senior centers in selected counties in Oregon, USA, for 48 weeks, followed by a 24-week post-intervention follow-up. Study process and outcome measures will be evaluated in accordance with the components of the RE-AIM framework that focus on Reach, Effectiveness, Adoption, Implementation and Maintenance.

**Discussion:**

This study will determine whether the evidence-based TJQMBB fall prevention program can be disseminated through a broad spectrum of community-based senior centers that often cater to low-income, underserved community-dwelling older adults at risk of falling. If shown to be both practically implementable and sustainable, the TJQMBB program will provide an effective, potentially low-cost, easy-to-implement intervention that could be used by public health practitioners and community-based organizations to address the problem of falls among older adults.

**Trial registration:**

ClinicalTrials.gov Identifier: NCT01854931

## Background

There is little debate about falls in older adults being a significant public health issue [1,2]. It is now well-established that (a) more than one third of community-dwelling adults aged 65 and older fall each year [1], (b) those who fall once are two to three times more likely to fall again [3], (c) most fractures among older adults are caused by falls [4], and (d) falls are the leading cause of injury deaths among individuals over 65 years of age [1,5-7]. All of these facts indicate that falling is a major threat to the independence and quality of life of seniors, in addition to being a significant burden on individuals, society, and national health systems [6,8-10]. From the perspectives of injury prevention and health promotion, this information underscores the need for greater dissemination and implementation of evidence-based fall prevention interventions [11].

Among various exercise programs that focus on increasing lower-extremity muscle strength and improving balance [12,13], Tai Ji Quan exercise has increasingly been demonstrated to reduce the incidence of falls among the elderly [14,15] and people with Parkinson’s disease [16] and is recommended by public health and healthcare experts as an exercise program to improve strength, gait and balance in older adults [3,9]. Thus, testing and translating new evidenced-based Tai Ji Quan programs into community practice to promote well-being and reduce the risk of injuries in older adults has become a public health and injury prevention priority [11,17].

The overarching objective of this study is to disseminate the evidence-based Tai Ji Quan: Moving for Better Balance (TJQMBB) program [18-20] through community senior centers that function to deliver services to America’s older adults [21]. Applying the RE-AIM framework [22], the primary aim of the study is to conduct a process and endpoint evaluation of the TJQMBB program to specifically assess the following relevant public health components (in order of importance):

1. Adoption – to examine the proportion of community senior centers that agree to adopt TJQMBB.

2. Reach – to examine the proportion and representativeness of the older adults who participate in the TJQMBB program.

3. Maintenance – to evaluate, upon completion of TJQMBB, the levels of sustained use of the program within the senior centers delivering the TJQMBB program and sustained participation in Tai Ji Quan exercises by participating individual older adults.

4. Implementation – to determine the degree to which the various TJQMBB program elements are delivered as intended.

5. Effectiveness – to examine the degree to which fall incidence is reduced and physical performance measures are improved.

A secondary aim of the study is to conduct a preliminary cost-effectiveness analysis of the TJQMBB program.

## Methods

### Study design overview

Within the RE-AIM framework [22], which is widely used for evaluating the public health impact of health promotion interventions and healthcare programs, we will evaluate the effect of our TJQMBB program via a 48-week, single-group, pre-and-post design with a 24-week post-intervention follow-up. The pre-and-post design allows us to assess the components of Adoption, Reach, Implementation and Effectiveness during the active 48 weeks of the intervention, whereas the 24-week post-intervention follow-up is planned to assess short-term program Maintenance.

### Study area

The dissemination area for this project is four counties in the state of Oregon, USA: Clackamas, Lane, Multnomah and Washington. The total population of these counties in 2012 was 2,045,327, of which 259,747 were persons 65 years old and over (per U.S. Census estimates), which accounted for 45% of the older adult population in the state. Per CDC data, these four counties ranked highest in Oregon in terms of the number of unintentional falls between 2004 and 2010 in individuals aged 65 and older, with the average annual (unintentional) fall fatality crude rate (per 100,000) being 85.8 in Clackamas, 73.2 in Lane, 89.9 in Multnomah, and 74.9 in Washington, compared with the national rate of 47.8 per 100,000 [23]. Within these counties, local community senior centers (defined below) constitute the primary locations where the TJQMBB program is disseminated and implemented.

### Dissemination adopters and population

In line with our pilot work [18], the program disseminating partners are community senior centers that provide health resources and social services, including meals and physical or recreational activities as part of a comprehensive service program to local community-living older adults. According to a list compiled from combined sources (*i.e*., local Area Agencies on Aging, county and state Senior & Disability Services, Oregon Recreation & Park Association), approximately 50 senior centers within the study area were potential candidates for our study.

Community-dwelling adults aged 65 and over living within the defined study area are the target population because this age cohort (a) is most at risk of falling [2,6,24] and (b) has been the target population for fall prevention in previous studies [12,13]. Participants are eligible to be enrolled in the TJQBMM program if they are (a) physically mobile (*i.e*., can walk with or without an assistive device), (b) without severe mental deficits, as determined by Mini-Mental State Evaluation (≥ 19) [25], (c) able to obtain a medical provider’s clearance, and (d) able to commit to the 48-week duration of the study.

### Recruitment

#### Senior centers

Eligible senior centers are initially contacted by research staff to solicit or confirm their interest in participating in this dissemination project. At the point of contact, a full description of the TJQMBB program is discussed with the center manager, program coordinator, or supervisor. When a center expresses interest in participating, a logistical plan that covers program/class promotion and set-up, size of the class, recruitment of participants, and class start-up date and scheduling is made jointly between the project staff and the participating center.

On the basis of available classroom sizes at the center, a maximum number of class enrollments is determined in consultation with staff at the participating center. The TJQMBB program advertisement and promotion is primarily done in collaboration with the participating center using sources such as monthly or quarterly newsletters, center websites, in-house flyers and class sign-up sheets, and word of mouth.

#### Participants

Prospective participants who respond to the promotion and/or sign up for the class at each center are contacted by telephone by the research staff to initiate a phone screening for initial eligibility. This is followed by an in-office visit where a full description of the program is given, consent forms are obtained, and study baseline measures are collected.

#### TJQMBB intervention

The TJQMBB program implemented was developed on the basis of early trials [14,26], modified in a community-based pilot project [18], and further refined and updated in two additional studies [16,19]. The full background of the evolution of this program is described elsewhere [20].

Per program protocol, each session begins with warm-up exercises (5 to 10 minutes) based on Tai Ji Quan movements, followed by the teaching and practice of core movements and subroutines that involve single forms and mini therapeutic movements (40 to 45 minutes), and ending with a simple set of breathing exercises (3 to 5 minutes). The core training protocol, which involves a set of simple but functional Tai Ji Quan-based actions, focuses on stimulating and integrating musculoskeletal and sensory systems via self-initiated movements such as weight shifting from foot to foot, unilateral weight-bearing, trunk rotation/flexion, ankle sways, and coordinated eye-head-hand movements, executed with integrated cognitive tasks [27]. The goal of the training is to improve postural stability and orientation, limits of stability, gait initiation and locomotion, gaze stability, movement symmetry and coordination, and lower-extremity strength. Chair-supported progressions, from completely seated, through sit-and-stand, to chair-assisted, are also included to: (a) meet the specific needs and performance capabilities of participants; and (b) train for functional activities involved in daily living.

A copy of a DVD with selected forms/movements of the program is distributed to all participants 4 to 6 weeks into the program. Participants are encouraged to use the material for additional home practice.

#### Class instructors

All instructors are chosen from local communities. In an effort to make the program more easily disseminable and generalizable, special efforts are made to recruit individuals who have little or no background in Tai Ji Quan or who currently work at participating centers giving instruction in other exercise modalities.

The first author trains all instructors via a two-day workshop at the Oregon Research Institute, using the training materials already developed. The training protocol includes a discussion of the theoretical underpinnings that were used to develop the program, findings from the efficacy trials and dissemination studies, detailed information and hands-on drills with the forms, variations of forms and routine exercises, mini therapeutic movements, and discussion of implementation- and teaching-related issues. Instructor training emphasizes consistency of delivery to ensure generalizability across different implementation centers. After instructors begin teaching, brief refresher training sessions are provided on a monthly basis.

### Measures

#### Demographics

General information that describes the social demographics of participating centers and their older adult clients is collected at baseline. Information on participating centers includes: types and number of services provided (*e.g*., meal services, education, physical activity); number of rooms devoted to physical activity; average class attendance across programs offered; and number of full- and part-time staff. Information on participants includes demographic and health characteristics, including age, gender, marital status, living situation, education, family income, race/ethnicity, existing medical conditions, use of medication, body weight, and body height. In addition, information regarding types of services received and frequency of visits to the service providers is also collected.

#### RE-AIM measures

Each component is operationalized per our study aim and assessed per procedures outlined by the RE-AIM developers (http://www.re-aim.org/) as described below.

Adoption is defined as the absolute number, proportion and representativeness of the senior centers that agree to adopt the TJQMBB program. Accordingly, we will calculate the adoption rate by dividing the number of centers that agree to participate (numerator) by the total number of centers approached within the dissemination geographic area (denominator). We document and report in detail on the following components: (a) total number of centers approached within the study area, (b) total number of centers that express interest in participating, (c) total number of centers that decline to participate (and reasons for declining), (d) total number of centers not responding to program promotion, (e) total number of centers participating, and (f) characteristics of participating centers. As part of the evaluation, the representativeness of participating centers will be determined by comparing them with the overall service characteristics of all senior centers in Oregon.

Reach is defined as the absolute number, proportion and representativeness of the older adults who participate in the TJQMBB program. This indicator will be assessed by first calculating the participation rate, using the number of eligible people qualified per study criteria and enrolled (numerator) divided by the total number of older adults who respond to the program promotion (denominator). In addition, we will also evaluate Reach by using the total number of older adults who attend regular activities at the participating centers as a denominator, which gives us a population index on the Reach component. In assessing the representativeness of our study population, we will compare information on demographics and health status of those who participated with those who met our recruitment criteria but who eventually, for any reason, declined to participate.

Maintenance is defined as the level of continued adoption of the TJQMBB program at the participating senior centers and sustained participation in Tai Ji Quan practice among the study participants after the completion of the 48-week active class implementation. At the center level, we will determine the number of participating centers that either continue offering the class or plan to adopt it in the future. At the individual participant level, we continue to collect data during the 24-week post-intervention follow-up with regard to participant Tai Ji Quan exercise status (*i.e*., continuing vs. not continuing) and with respect to time (length of practice sessions) and frequency (number of sessions per week).

Implementation is defined as the degree to which instructors deliver the various intervention elements as intended. In this regard, measures of fidelity from our previous studies of this program are used [16,19]. Specifically, the extent to which the program is implemented per the pre-specified requirements is assessed in relation to the extent to which class instructors successfully implemented the various program components, including (a) delivering twice-weekly 60-minute sessions over a 48-week period (verified by project staff), (b) adhering to the teaching emphases and training protocols (verified by a checklist), and (c) achieving participation and retention rates of 70% or better (verified by class attendance sheet). The components in adherence to the teaching and training protocols focus primarily on delivery (*i.e*., teaching emphases and movement execution), integration (*i.e*., blending of forms, variations of forms, and mini therapeutic movements), and actual practice time (40 to 45 minutes spent on core movements). The first author or an experienced peer instructor conducts in-class fidelity checks at least every two months.

As part of the evaluation for our secondary study aim, the implementation component will include documenting project implementation costs. Specifically, direct costs associated with implementation of the intervention will be documented, including (a) participant recruitment costs (administration staff costs, printed materials for program promotion, advertisements), (b) instructor liability insurance costs, (c) instructor salary costs, (d) classroom rental costs in the local community, (e) cost of the initial two-day training and refresher courses for instructors, as well as in-class fidelity checks, and (f) other expenses related to program promotion and supplies (*e.g*., water bottles, DVDs).

Effectiveness in this study is defined as the degree to which fall incidence is reduced and physical performance measures have improved. Specifically, falls are assessed using fall counts, recorded by each participant in a daily ‘fall calendar’. Participants are instructed to record any fall event and to indicate whether the fall caused them to seek medical attention. A fall is defined as ‘when you land on the floor or the ground, or fall and hit objects like stairs or pieces of furniture, by accident’. A fall is considered ‘injurious’ if the fall resulted in fractures, head injuries, sprains, bruises, scrapes, or other serious joint injuries, or if the individual sought medical care. As in our previous studies [14,16,19], falls are monitored for all participants throughout the 48-week intervention, or until the date they withdrew from the study.

In addition, physical performance-based measures are collected. These include (a) Timed Up & Go test [28], (b) Multi-Directional Reach Test (in three directions – forward, backward, lateral) [29], (c) chair stands [30], and (d) 50-foot speed walk.

#### Class attendance

Class attendance is recorded for each participant at each class session and evaluated monthly by research staff. The attendance data will be tallied at the end of the class intervention across all participating centers to derive an average class attendance for the study.

#### Analysis plan

Descriptive statistics will be presented for all outcome measures. The fall incidence rate will be determined as the number of falls that occurred during the intervention period divided by the total follow-up time in months (person-months of follow-up). Paired t-tests are planned to examine change in functional outcomes of participants from baseline to program termination at 48 weeks. Because senior centers are the primary sampling unit (PSU) in this study, a clustering data structure (*i.e*., participants are nested within centers) can result. To account for potential clustering effects, outcome data will be analyzed with sampling weights and a clustering variable (PSU) that allows correction of the correlation between participants within centers and standard errors of estimates.

Average cost-effectiveness will be determined [31] in which the cost-effectiveness ratio will be expressed as cost per fall prevented, calculated by dividing the direct cost of the intervention by the total number of falls prevented by the Tai Ji Quan intervention. There will be no out-of-pocket expenses paid by participants. Program development costs will not be included in the cost-effectiveness analysis. Costs after the first year of the study (2008 as baseline values) will be discounted at 3% annually.

#### Study status

Recruitment of senior centers and individual participants began on March 1, 2012. The first class of the TJQMBB intervention began on April 3, 2012. The overall dissemination project is on-going and is expected to run through May 2015, with data analysis on components of RE-AIM and cost-effectiveness beginning immediately after that.

## Discussion

### Significance

In an effort to highlight effective fall prevention programs, the CDC’s Injury Center has created a resource for community organizations, seniors, and caregivers entitled ‘Preventing Falls: What Works. A Compendium of Effective Community-based Interventions from Around the World’ [3]. The compendium presents 14 randomized controlled intervention trials, including Tai Ji Quan, with proven effectiveness in reducing falls among older adults. Although the resource gives public health practitioners and community-based organizations relevant details about a variety of interventions that use exercise, home modification, or multifaceted strategies, many of these evidence-based programs have not undergone rigorous real-world (dissemination) evaluation to validate their use and adoption in communities. Thus, there is a pressing need to translate and disseminate evidence-based exercise programs to community service providers that serve older adults at increased risk of falling. This study, therefore, not only deals with the significant public health concern over falls in older adults but, more importantly, it addresses the significant research-to-practice gap in the field of injury prevention by disseminating an evidence-based TJQMBB program through community senior centers.

### Innovation

To our knowledge, this is the first Tai Ji Quan study to adopt a community-based approach to evaluate an evidence-based program that has already undergone research trials and initial adaptations in local community settings. Such a study is sorely needed given the consequences of falling for older adults, the accumulated evidence of Tai Ji Quan efficacy in reducing risk of falling, and the need for translating evidence-based research into community-based programs that are accessible through local senior and adult activity centers. Second, the effort to evaluate the success of the program via the well-established RE-AIM framework is also novel, providing a theoretically sound approach for evaluating the program’s potential for translation from research to community practice. Finally, the emphasis on training existing instructors and/or staff in the implementation centers is also a novel approach in that it will both facilitate class teaching and program sustainability and potentially reduce program costs. Also, it is well documented that sustaining important public or grant-funded services after initial funding is terminated is a major public health challenge. Our study responds to this challenge in that it involves significant collaboration with community adopters, allowing us to formally evaluate the sustainability of the program over time at the service provider level.

### Potential impact

The effort of this study to move from research to community-based utilization has significant potential to serve the public health need to address the falls problem in older adults. If successfully implemented with demonstrable outcomes, this work will allow us to fill an important knowledge gap regarding the feasibility of wide community dissemination, program adaptations and scalability, and the economic point at which this low-tech TJQMBB program may be offered by senior service providers, program funders, policymakers, and healthcare planners to address the increasing need for efficacious, but cost-effective and community-based, falls prevention programs.

### Limitations

Two limitations of this study should be noted. First, this study evaluates the program in one state, which may limit its generalizability to other states with varying demographic characteristics of older adult populations. Similarly, the study involves senior centers that are located primarily in urban areas, which may influence the adoption and implementation potential of the program in rural areas that may have different characteristics, such as constrained resources, small space, or short staffing. Second, the lack of a comparison or control group because of the pre-post single-group design in this study should be kept in mind when assessing the net gain of program effectiveness.

## Conclusions

Fall prevention programs, no matter how effective, cannot enhance public health unless they are translated and disseminated in an appropriate and timely manner to older adults and the organizations that serve them. Currently, few evidence-based fall prevention programs have been systematically and rigorously disseminated and evaluated in community practice. This study addresses this significant research-to-practice gap; and if the study objectives are successfully achieved, new dissemination knowledge can be gained in terms of developing successful strategies and transferable methods for disseminating and implementing evidence-based fall prevention programs for agencies that serve the target population. Thus, the potential impact of the study on the field of fall prevention for older adults is very high.

### Ethical considerations

The study protocol was approved by the Institutional Review Board of Oregon Research Institute (IRB Registration No. 00000278).

## Abbreviations

TJQMBB: Tai Ji Quan: moving for better balance.

## Competing interests

The authors have no competing interests to declare.

## Authors’ contributions

FL and PH obtained funding for this study and contributed to the design of the study and writing of this manuscript. Both authors have read and approved the final manuscript.
